# An innovative synthesis approach for Na A. Zeolite: A new pathway for enhanced performance, wastewater treatment, and antibacterial applications

**DOI:** 10.1371/journal.pone.0332132

**Published:** 2025-09-11

**Authors:** Mutairah S. Alshammari, Amro H. Mohamed, Samah A. Mohamed, Hussein M. Ahmed

**Affiliations:** 1 Department of Chemistry, College of Science, Jouf University, Sakaka, Aljouf, 72341, Kingdom of Saudi Arabia; 2 Housing and Building National Research Center (HBRC), Sanitary & Engineering and Environmental Institute, Giza, 12613, Dokki, Egypt; Universiti Teknologi Petronas: Universiti Teknologi PETRONAS, MALAYSIA

## Abstract

Wastewater treatment is essential for protecting water resources and public health. Zeolite-based adsorbents offer an effective and sustainable solution for this purpose, providing high selectivity and regeneration potential. Zeolites are inorganic, highly crystalline, micro-porous materials composed of aluminotecto-silicates (SiO₄ and AlO₄ tetrahedral). Synthetic zeolites are commercially favored over natural ones due to their higher purity, crystallinity, and uniform pore size. Na A. zeolite (NaAZ) is a type of synthetic zeolite widely used in various applications, including wastewater treatment, due to its excellent adsorption and ion-exchange properties. This study focus on synthesize zeolite A from meta-kaolinite using a wet chemical method. The synthesis involves a hydrothermal process in which chemical reagents are mixed in an aqueous medium and heated under controlled conditions. The resulting (NaAZ) was characterized using Brunauer-Emmett-Teller (BET) surface area analysis, Fourier Transform Infrared Spectroscopy (FT-IR), X-ray Diffraction (XRD), Scanning Electron Microscopy (SEM), and Energy Dispersive X-ray Spectroscopy (EDX). This study evaluates the synthesized (NaAZ) for the removal of chemical oxygen demand from synthetic wastewater. Various parameters affecting adsorption such as contact time pH, temperature, and adsorbent dosage were investigated. The optimized conditions were then applied to real wastewater, and the material was further tested for its antitoxic and antibacterial properties. The in vitro antibacterial activity of NaAZ was assessed against both Gram-positive bacteria (Bacillus subtilis ATCC 6633, Staphylococcus aureus ATCC 6538, Enterococcus faecalis ATCC 19433) and Gram-negative bacteria (Escherichia coli ATCC 25922, Enterobacter aerogenes ATCC 13048, Pseudomonas aeruginosa ATCC 15442). Under optimal conditions contact time (40 min), pH (6–7), and adsorbent dosage (0.25 g) the removal efficiencies for COD, TSS, TKN, and PO₄^3^⁻ were 90.69%, 90.41%, 73.75%, and 68.85%, respectively.

## Introduction

The extremely fast growth of the world population in the last century, in addition to the industrial revolution, reflected in a considerable rise in both fresh water consumption and wastewater production. Fresh water demand has already exceeded supply, and currently special treatment is more and more often required in order to obtain drinking water of high quality as well as to produce environmentally acceptable effluents. Water from natural, industrial, municipal or agricultural origin may contain variety of suspended, dissolved, colloidal or emulsified organic and inorganic impurities [1,2].

Several forms of wastewater treatment have been developed over the decades, to remove contaminating elements from wastewater, before their disposal. Techniques such as chemical precipitation, ionic flotation, electro-dialysis and biological treatment are efficient in the treatment of effluents, but they have some disadvantages such as the generation of toxic waste that requires adequate storage, high demand for chemical reagents, high energy cost, and limited efficiency to remove contaminants with low concentration. In contrast, the adsorption technique is promising and possesses several advantages over other techniques, which justifies its application in the treatment of effluents. One of the great advantages of the technique is its versatility, as it allows the use of various materials as adsorbents, from activated carbon to industrial wastes [1].

Several materials have been used as adsorbents to remove contaminants from water. Activated carbon, bio-char, clay minerals, nano-materials [3], agro-waste adsorbent and natural waste materials. These materials possess the necessary features to be used for this purpose, high surface area, negative electric charge, and meso-porous materials. However, some disadvantages limit their use on a large scale. Activated carbon and bio-char are products of the calcination of organic matter, corn straw, and rice husk, in an anaerobic atmosphere [1].

Zeolite is inorganic material, highly crystalline, micro-porous, and structure component is alumino-silicates (SiO_4_ and AlO_4_ tetrahedral) as ring structure connected by oxygen atoms at their corner side [4]. The structure of zeolites generates uniformly sized interconnected micro-pores and [5–7]. Cations sorption is determined by the uncompensated negative charge, resulting from the substitution of metals by Al in the tetrahedra. Cation exchange capacity (CEC) is a measure of the number of cations per unit weight available for exchange, usually expressed as milli-equivalents per gram of material. The sorption capacity and selectivity for water and other molecules are defined by zeolite porosity, pore size distribution, and specific surface [2]. Due to their unique properties, zeolites have found a broad range of industrial applications as catalysts, adsorbents, molecular sieves and ion exchangers with a growing global market. They have been used in a large number of water treatment processes such as water softening and purification from ammonia, heavy metals, radioactive species, dissolved or emulsified organic substances, toxic anions, odor and solids [2].

Zeolites are usually prepared from dense gels containing silica and alumina species at elevated temperatures in a relatively expensive process [4,6,7]. Na A zeolite is one of the simplest synthetic zeolites with elemental ratio of (Si:Al:Na). Na A zeolite 1:1:1 [8]. This structure is responsible for its high surface area and porosity, making it an effective adsorbent [8]. Na A zeolite is effective at adsorbing organic molecules and inorganic ions. They are widely used in water treatment for the removal of pollutants such as ammonium (NH₄⁺), and organic pollutants. Adsorption capacity is influenced by factors like the particle size, ionic composition, and pH of the solution. Na-zeolite can be regenerated and reused, making it a sustainable and cost-effective option for long-term use. Na A zeolite adsorbs various pollutants, including organic compounds, dyes, and suspended solids, due to its high surface area and micro-porous structure [8,9].

Aim of this study to synthesize NaAZ using the wet chemical method and evaluate its efficiency in treating contaminated wastewater. Characterize the synthesized zeolite to investigate the structural, morphological, and chemical properties of the synthesized (NaAZ) using various analytical techniques x-ray diffraction (XRD), scanning electron microscopy (SEM), Fourier transform infrared (FT-IR), energy dispersive x ray (EDX), and BET surface area analysis. Assess the adsorption properties to evaluate the adsorption efficiency of NaAZ for removing specific contaminants from synthetic wastewater samples (COD). Examine regeneration and reusability to investigate the regeneration potential of NaAZ for repeated cycles of wastewater treatment and assess its sustainabilit**y** as a treatment material. To identify the key factors (pH, contact time, dosage, and initial concentration) that influences the efficiency of NaAZ in treating wastewater. Evaluate environmental impact and cost-effectiveness: to assess the environmental impact, cost-effectiveness, and practical applicability of using NaAZ in large-scale wastewater treatment, with a focus on potential industrial applications. By achieving these goals, the study aims to contribute valuable insights into the use of NaAZ as a promising material for improving wastewater treatment technologies.

## Materials and methods

### Synthesis of NaAZ using wet chemical method

The Sodium hydroxide (NaOH, MERCK, 99.8%, 3.0 M) in water to create a strong alkaline solution, add the silica source (sodium meta-silicate, LOBA, 94%) to the solution and stir until dissolved, add the aluminum source (Al₂(SO₄)₃, LOBA, 99%) to the mixture, ensuring thorough mixing. Once the components are mixed, the solution may appear as a gel. The gel is typically stirred for 2.0 h, to ensure the complete dissolution of the reactants. The gel is then transferred into a dryer for heated at a temperature 80**°C** for 12 h, the gel undergoes crystallization, resulting in the formation of Na-A zeolite. After the hydrothermal process, the sample is cooled to room temperature. The zeolite crystals are filtered washed several times with distilled water to remove excess sodium hydroxide and other impurities. The washed zeolite is then dried in an oven at 105°C to [2–4] h to obtain Na-A zeolite [10]. Sigma-Aldrich Co., St. Louis, Missouri, USA) was utilized as the standard reference for all bacterial Strains for this test specifically (*Bacillus subtilis ATCC6633; Staphylococcus aureus ATCC6538;* Enterococcus faecalis ATCC 19433*) as gram positive* and *(Escherichia coli ATCC 25922; Enterobacter aerogenes ATCC13048;Pseudomonas aeruginosa ATCC15442)* as gram negative. Tryptic soy broth (Merck, Germany), Buffer Solution (KH_2_PO_4,_ Merck, Germany), sterile deionized water grade (B), plate count agar media (Merck, Germany). This study was carrying out in Housing and Building National Research Center, Sanitary and Environmental Institute, Egypt.

### Characterization of NaAZ

When characterizing Na-zeolite various analytical techniques such as Brunauer-Emmett-Teller method (BET)” (Quantachrome Instruments, USA), “Fourier Transform Infrared Spectroscopy” (FT-IR 8400S, Shimadzu, Tokyo, Japan) in the 400–4000 cm^-1^ spectrum range, “X-ray diffraction (XRD)”, “Scanning Electron Microscopy (SEM)”, “Energy Dispersive X-ray spectroscopy (EDX)” (SEM, EDX model Bruker’s of QUANTAX, US) can provide crucial information about their composition, structure, surface properties, and morphology. The Phase characterization was carried out by “X-ray diffraction (XRD)” using a diffractometer; model XDS 2000 with Ni-filtered Cu Ka radiation [11].

### Adsorption study experiment

Prepare stock solution from COD parameter, this solution using as synthetic wastewater for evaluated the Na zeolite A. Batch experiments were used to measure the removal of chemical oxygen demand (COD) at varied concentrations ranging from 100 to 1000 mg/L. Each test involved filling 1.0 L at a solid/solution ratio of 0.1 g/L. The mixture was then agitated for 150 rpm, 30 min at 20 °C, the concentration of COD in each sample was measured after procedure. The samples were measured using the spectrophotometer to measure very low concentrations. Equations (1, 2) were used to determine the amount of pollutants adsorbed at equilibrium (q_e_) and the percentage removed (R %) [11].


$$\text{R}\%=\frac{\left(Ci-Ce\right)}{Ci}\;x\;100$$ (1)

$$\text{qe}=\frac{(Ci-Ce)V}{W}$$ (2)

Where q_e_ (mg/g) represents the amount of pollutants adsorbed at equilibrium, V (L) is the volume of the solution, W (g) is the mass of the nanoparticles used, C_i_, and C_e_ (mg/L) represents COD concentrations at initial and equilibrium conditions, respectively.

### Influence of contact time

During varied contact times the removal of COD by (NaAZ) was investigated. The studies were conducted at 20 °C. The adsorbent dose was 0.1 g, and contact times were 10, 20, 30, 40, 50, and 60 min. The adsorbent was combined with a synthetic aqueous solution at a concentration of 500 mg/L, The aqueous solution volume utilized in the experiment was 1.0 L at 150 rpm [12].

### Effect of dose on adsorption profile

The investigation of the effect of adsorbent dose on adsorption capacity is also part of batch studies. The aqueous solution volume utilized in the experiment was 1.0 L at 150 rpm, the contact period was 40 min, and the initial concentration of COD was 500 mg/L. The adsorbent was mixed with an aqueous solution at various dosages (0.05, 0.1, 0.15, 0.2, 0.25 and 0.3 g) in the combined system [12].

### Effect of initial concentration

The experiment was conducted by creating different COD concentrations and holding the temperature constant at 20 °C. It was investigated how the initial concentration of COD affected the effectiveness of adsorption [13]. The adsorbents were mixed with an aqueous solution at concentrations of (100, 200, 300, 400, 600 and 1000) mg/L with the same 40 min contact time and dose of 0.3 g. The experiment used 1.0 L of a synthetic aqueous solution rotating at 150 rpm [12].

### Effect of pH

The pH is the most critical parameter affecting any adsorption studies due to their interference in the solid–solution interface, affecting the charges of the active sites of the adsorbents and the COD behavior in the solution(Sidkey 2020). The effect of pH on the removal of COD in the solution has been established and considered an important parameter affecting the performance of the adsorption process. At varied pH levels (3, 6, and 9), removing of COD from the aqueous solution was conducted with a constant dosage of 0.1 g at 150 rpm/50 min, and 500 mg/L [12].

### Breakthrough curve in adsorption studies

Column studies simulate real wastewater treatment systems and provide insights into the long-term performance and reusability of Na-A zeolite. Pack a column with Na-A zeolite. Pass contaminated water through the column at a controlled flow rate. Monitor the effluent for concentrations of pollutants. Measure the removal efficiency at different time (5, 10, 20, 30. 40, 50, and 60) min [14], factors affecting breakthrough curve on removal of NaAZ such as surface area, bed height, and flow rate of wastewater. Flow rate of sample in column system is 2.5 ml/min, bed height (50 cm). The effectively adsorption of NaAZ for COD was plotted as C_t_/C_0_ vs. time (t), where: **C**t = Effluent COD concentration (mg/L), **C**₀ = Initial COD concentration (500 mg/L).

### Isotherm models

#### Langmuir isotherm model.

Adsorption isotherms, which are typically the ratio between the amount adsorbed and that was left in solution at equilibrium at a specific temperature, are used to describe equilibrium studies that give the capacity of the adsorbent and adsorbate [15]. The Langmuir model is predicated on the hypothesis that maximal adsorption happens in the presence of a saturated monolayer of solute molecules on the adsorbent surface, the adsorption energy is constant, and there is no adsorbate molecule migration in the surface plane. The Langmuir isotherm model implies that physical factors drive monolayer sorption. The Langmuir isotherm is given by Equations (3, 4 and 5):


$$\text{qe}=\frac{\text{C}.\text{K qmax}}{\text{1}+\text{KL}}$$ (3)

$$\frac{\text{Ce}}{\text{qe}}=\frac{\text{1}}{\text{qmax }.\text{ KL}}+\frac{\text{Ce}}{\text{qmax}}$$ (4)

$$\text{RL}=\frac{\text{1}}{\text{1}+\text{Co}.\text{KL}}$$ (5)

where q_max_ and K are the Langmuir constants, where qe is the dose of COD adsorbed on a specific dose of adsorbent (mg/g), C_e_ is the equilibrium concentration of the solution (mg/L) and q_max_ is the maximum dose of COD concentration required to form a monolayer (mg/g). The values of q_m_ and K can be determined from the linear plot of C_e_/q_e_ versus C_e_ [16]. R_L_ is separation factor calculated through Langmuir model.

#### Freundlich isotherm model.

The Freundlich isotherm model proposes that many sites with various adsorption energies are involved in the empirical relationship that describes the adsorption of solutes from a liquid to a solid surface. The system’s characteristics K_F_ and n are the indicators of the adsorption capacity and adsorption intensity, respectively. The Freundlich model’s capacity to match the experimental data was investigated. The intercept value of K_F_ and the slope of n were calculated for this scenario using the plot of log C_e_ vs. log q_e_. The Freundlich isotherms appear when the surface is heterogeneous and the absorption is multilayered and bound to sites on the surface. The Freundlich isotherm is given by Equation (6):


$$\text{log}\text{qe}=\text{log}\text{KF}+\frac{\text{1}}{\text{n}}\text{log}\text{Ce})$$ (6)

where K is the Freundlich equilibrium constant (mg/g), 1/n = Intensity parameter C_e_ = Equilibrium concentration of adsorbate, q_e_ is the dose of solute adsorbed. Freundlich model with linear plotted log q_e_ versus log C_e_ shown in Equation (6) [14].

The constants K_f_ and 1/n are produced via the Freundlich formulation in a linear form. Freundlich isotherm model assumes non-ideal adsorption on heterogeneous surfaces in a multilayer coverage. It suggests that more robust binding sites are occupied first, followed by weaker binding sites. In other words, as the degree of site occupation increases, the binding strength decreases [17].

### Kinetic study

It was possible to characterize the kinetics for each adsorbent, using pseudo-first and pseudo-second-order kinetic models. The pseudo-first-order kinetics follows the Lagergren model expressed by Equation (7):


$$\text{Log }(q_{eq}-q_{t})\;=\;{\text{log}q}_{eq}-K_{\text{1}}\;.\;t/\text{2}.\text{303}$$ (7)

where q_t_ is the adsorbed dose of COD (mg/g) in t time (min) and k1 is the pseudo-first-order constant (min^-1^).

Through linear and angular constant of log graphic (q_eq_ – q_t_) in the function of time, q_eq_ and k_1_ can be calculated, respectively. Comparing the experimentally obtained values for q_eq_ calculated by pseudo- second-order kinetics by Equation (8):


$$t/q_{t}=\text{1}/K_{\text{2}}{q_{eq}}^{\text{2}}+t/q_{eq}$$ (8)

where k_2_ is the pseudo-second-order constant (g/mg. min) obtained by calculation of linear coefficient and q_eq_ is calculated through angular coefficient [14,18].

To evaluating the goodness of fit for non-linear kinetic models (pseudo-first-order, and pseudo-second) in adsorption and biodegradation processes, error analysis is essential such as Sum of Squared Errors (SSE). (q_e_exp- q_e_ cal)^2^


$$\text{SSE}=\sum_{i=1}^{n}{\left({\text{q}}_{\text{e}}\text{exp}-{\text{q}}_{\text{e}}\text{ cal}\right)}^{\text{2}}$$ (9)

Where q_e, exp_, is the experimental adsorption capacity and _qe, calc_ is the calculated value from the model. Lower SSE values indicate a better fit of the model to the experimental data, and highly sensitive to large deviations.

### Regeneration and reusability studies of NaAZ for wastewater treatment

Regenerating zeolite allows its reuse for multiple cycles in wastewater treatment while maintaining high adsorption efficiency. Various studies and practical applications have demonstrated the effectiveness of regenerated zeolite in removing contaminants such as COD. Over time, zeolite becomes saturated reducing its efficiency; **r**egeneration is the process of restoring its adsorption capacity, allowing repeated use and improving cost-effectiveness. Several techniques can be used to regenerate zeolite. Thermal regeneration (Calcination), heating zeolite at 600°C to removes organic contaminants by combustion, achieve near-complete regeneration but are energy-intensive. Effective for removing adsorbed organics and volatile pollutants. To assess the economic feasibility of NaAZ for wastewater treatment, studies on its regeneration and reusability are essential. This provides an understanding of the cost-effectiveness and sustainability of NaAZ in wastewater treatment applications. Zeolites can be regenerated by washing them with HCl solutions (1.0 N) to replace the adsorbed ions with fresh ones. After treating wastewater, wash the NaAZ with a regeneration solution. Reuse the zeolite in successive cycles of wastewater treatment. Regenerate the zeolite after a five cycles by flushing the column with a regeneration solution HCl (1.0 N). A five-cycle regeneration study evaluates the adsorption capacity, efficiency loss, and structural stability of zeolite when treating COD pollutants.

### Case study- using NaAZ for treatment of real raw wastewater

Wastewater samples were collected as in Giza, Egypt. Samples were obtained from raw wastewater. The characteristics of the wastewater indicated that it was relatively strong, as evidenced by high levels of total suspended solids (TSS), biological oxygen demand (BOD_5_), Total kjledahl nitrogen (TKN), chemical oxygen demand (COD), and phosphate. Physicochemical analyses were conducted in accordance with standard methods for the examination of water and wastewater [19]. Optimal conditions are applied to sewage samples: a contact time of 40 min, a dosage of 250 mg, pH of 6–7, at room temperature of 20–25°C. The results obtained are used to determine the efficiency of the adsorbent and calculate the treatment ratio according to the Equation (1).

### Evaluation of NaAZ as antibacterial adsorbent

Weight 1.0 g of Na A. zeolite, the material must settle after shaking so that no specimen interferes with the retrieval and counting techniques. Untreated and treated specimen is required. Prepare one sterile 250 mL screw-cap Erlenmeyer flask for each treated and untreated specimen, and one “inoculum only” sample for the series being run. Add 50 ± 0.5 mL of working dilution of bacterial inoculum prepared in 10.2 to each flask.

### Bacterial strains

Sigma-Aldrich Co., St. Louis, Missouri, USA) was utilized as the standard reference for all bacterial Strains for this test specifically (*Bacillus subtilis ATCC6633; Staphylococcus aureus ATCC6538;* Enterococcus faecalis ATCC 19433*) as gram positive* and *(Escherichia coli ATCC 25922; Enterobacter aerogenes ATCC13048;Pseudomonas aeruginosa ATCC15442)* as gram negative.

### Preparation of bacterial inoculum

**Fig 1:**
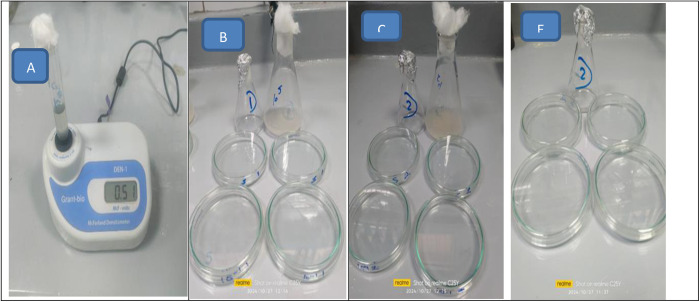
Inoculum flask with 108 cfu/ml with maccfland (A), Inoculum flask with after dilutions for assessment antibacterial activity of NZ(B), Inoculum flask with after dilutions for assessment antibacterial activity of AZ (C), AZ Activated Zeolite tested sample (E).

Grow a fresh 18 h shake culture of *Escherichia coli* in sterile Tryptic Soy Broth at 35 ± 2°C prior to performing the test. Dilute the culture with the sterile buffer solution until the solution has an absorbance of 0.28 ± 0.02 at 475 nm, as measured spectrophotometrically. This has a concentration of 1.5–3.0 × 10^8^ CFU/ml. Dilute appropriately into sterile buffer solution to obtain a final concentration of 1.5–3.0 × 10^5^ CFU/ml. This solution will be the working bacterial dilution. Fig 1 shows the predation of tested samples and bacterial inoculums.

### Toxicity Test procedure

NaAZ is as the control was each added to1.0 L of autoclaved distilled water individually. The test was then conducted, and the results were reported in accordance with [20]. Sample was prepared, examined and result recorded according to (ASTM E2149-20).This test determines the antimicrobial activity of a treated specimen by shaking samples in a concentrated bacterial suspension for a one hour contact time. The suspension is serially diluted both before and after, contact and cultured. The number of viable organisms from the suspension is determined and the percent reduction is calculated by comparing retrievals from appropriate controls. Employing the tested Microbes *Vibrio fischeri* and Microtox analyzer 500, during testing, the bacteria were exposed to different solutions, Along with standard solutions and control samples. The bacteria’s reduction in light emission was assessed using the Microtox Omni Azur software, the data were recorded and the EC_50_ (concentrations generating a 50% reduction in light) is computed [21,22].

## Results

### Physical properties and BET analysis of NaAZ

**Fig 2:**
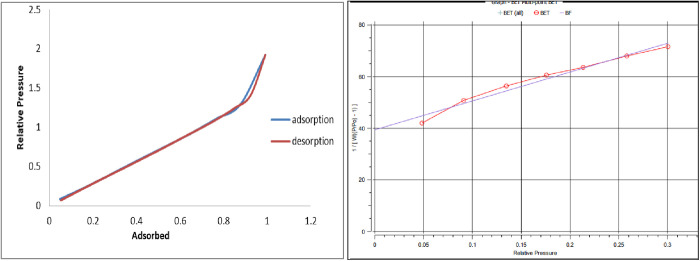
BET analysis of NaAZ.

Based on its structure and composition, NaAZ has high ionic conductivity and high ion-exchange capacity that facilitate its introduction in various applications such as detergency, desiccation, adsorption, separation, and ion exchange [23]. BET analysis measures the surface area and porosity of materials by using nitrogen adsorption-desorption isotherms. NaAZ is a highly porous material with a large surface area and a regular structure of micro-pores, which allows it to adsorb a wide range of ions and molecules, as shown in Fig 2, and Table 1: Due to its large surface area and porosity, NaAZ can adsorb significant amounts of contaminants. NaAZ selectively removes heavy metals, ammonium, and organic compounds, making it versatile. The BET surface area of NaAZ is typically high, due to its porous structure. The total surface area and pore volume can give insights into its accessibility and porosity.

**Table 1:** Physical properties and BET analysis of NaAZ.

Appearance	Orange
Smell	None
Plasticity	None
Humidity at drying 110°C	10.3
Solubility in water	None
pH value	6.5–7.5
Density	2.23 g.cm^-3^
Molecular Weight	28.0134 g
Specific surface area (BET)	22.98 m^2^/g
Average Pore radius	2.8952 nm
Cross Section Area	16.2 Å²/molec

### X-ray diffraction (XRD)

**Fig 3:**
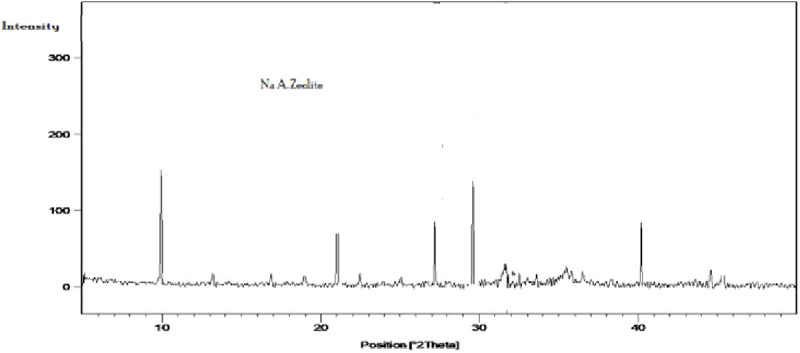
X. ray diffraction patterns of NaAZ.

The XRD pattern of the synthesized product Fig 3 displays sharp diffraction peaks at 2θ = 10.22°, 21.74°, 27.08°, 30.16°, and 40.88°, corresponding to the (100), (200), (321), (400), and (420) lattice planes of LTA-type NaAZ. The excellent match with the crystalline form and the negligible amorphous background confirm the successful crystallization and high purity of the NaAZ [24]. The patterns confirm the crystallization of NaAZ, which is pure, while some hydroxyl-sodalite was identified in the synthesis product of the sample treated with 3.0 N NaOH concentrations, similar observations were given by many authors to indicate the formation of hydroxysodalite at higher alkalinities [8]. Table 2 shows the X-ray fluorescence analysis of NaAZ. The XRF data show clear increase in the Si is 65%, Al is 17.5%, Na is 6.5%, indicating the composition of NaAZ, as (Si:O:Al), We can also notice that the Wt % ratio of SiO_2_/Al_2_O_3_ shows slight increase from 3.7 [25].

**Table 2:** Chemical composition of the NaAZ.

Chemical composition	Amount %
SiO_2_	65
Al_2_O_3_	17.5
Na_2_O	6.5
Others	11

### The scanning electron microscopy (SEM)

**Fig 4:**
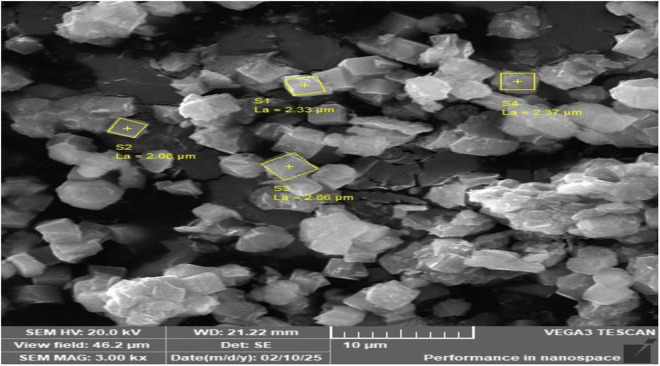
SEM micrographs of NaAZ.

The Fig 4 show well-developed crystalline consists of characteristic cubic-shaped crystals of NaAZ type with sharp edges and average grain size of less than 70 *µ*m. The SEM results of crystal show aggregates of parallel oriented and partially reacted plates of meta-kaolinite as well as some dispersed NaAZ cubes. SEM provides high-resolution imaging of the sample surface, giving information on the morphology, surface texture, and particle size. The particle size of NaAZ can affect the efficiency of pollutant removal. Smaller particles generally provide more surface area for adsorption.

### Energy dispersive X-ray spectroscopy (EDX)

**Fig 5:**
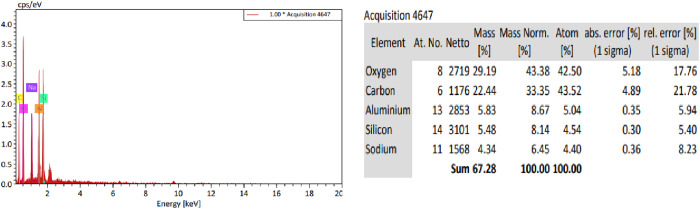
EDX and percentage constituents of NaAZ.

The EDX analysis of the synthesized NaAZ provides detailed insights into its elemental composition, confirming the successful incorporation of sodium (Na), aluminum (Al), silicon (Si), and oxygen (O) within the crystal framework. The elemental ratios closely align with the theoretical stoichiometry of NaAZ, supporting the successful synthesis and structural integrity of the product. Fig 5 shows EDX analysis of the NaAZ reveals the elemental composition of the synthesized product, showing the presence of oxygen (29.19 wt%), silicon (5.48 wt%), aluminum (5.83 wt%), and sodium (4.34 wt%). These values are consistent with the typical chemical composition of NaAZ, confirming the successful formation of its aluminosilicate framework. Zeolites are mainly constituted by Al, O, and metals including Ti, Sn, Zn, and others [26].

### Fourier transform infrared spectroscopy (FT-IR)

**Fig 6:**
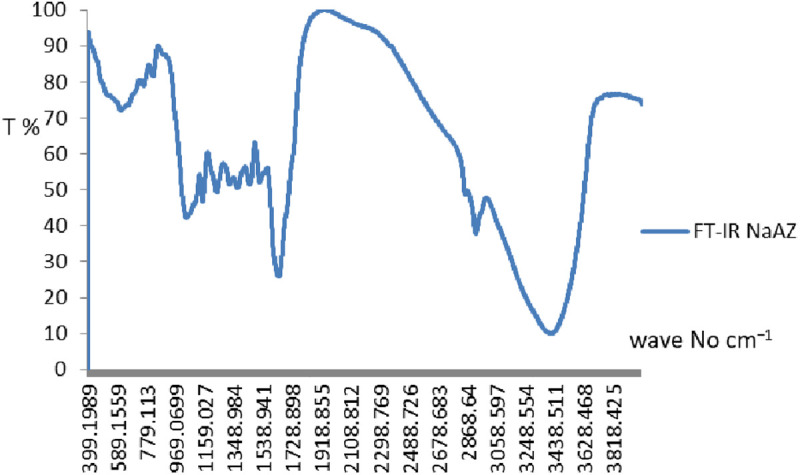
FT-IR for the NaAZ.

Infrared spectroscopy measures the absorption of infrared light by the sample, revealing information about functional groups and molecular vibrations. The infrared spectrum of NaAZ is given in Fig 6. The FT-IR results show typical bands at 3421 cm^−1^, 1166 and 1080 cm^−1^ representing the Si–O stretching. Characteristic peaks related to the Si–O and Al–O stretching vibrations, and possibly adsorbed water or hydroxyl groups [27,28]. Meta-kaolinite is formed with intense bands at 1080 cm^−1^, as the major feature. For meta-kaolinite, the disappearance of the 530 cm^−1^ band indicates the loss of Al[O(OH)]_6_ [29,30]. Bands associated with water adsorbed by the zeolite pores due to the presence of van der Waals interactions between the hydroxyl groups in the zeolite structure related to H_2_O and the positive charge on the surface of Na^+^ [31].

### Influence of contact time

**Fig 7:**
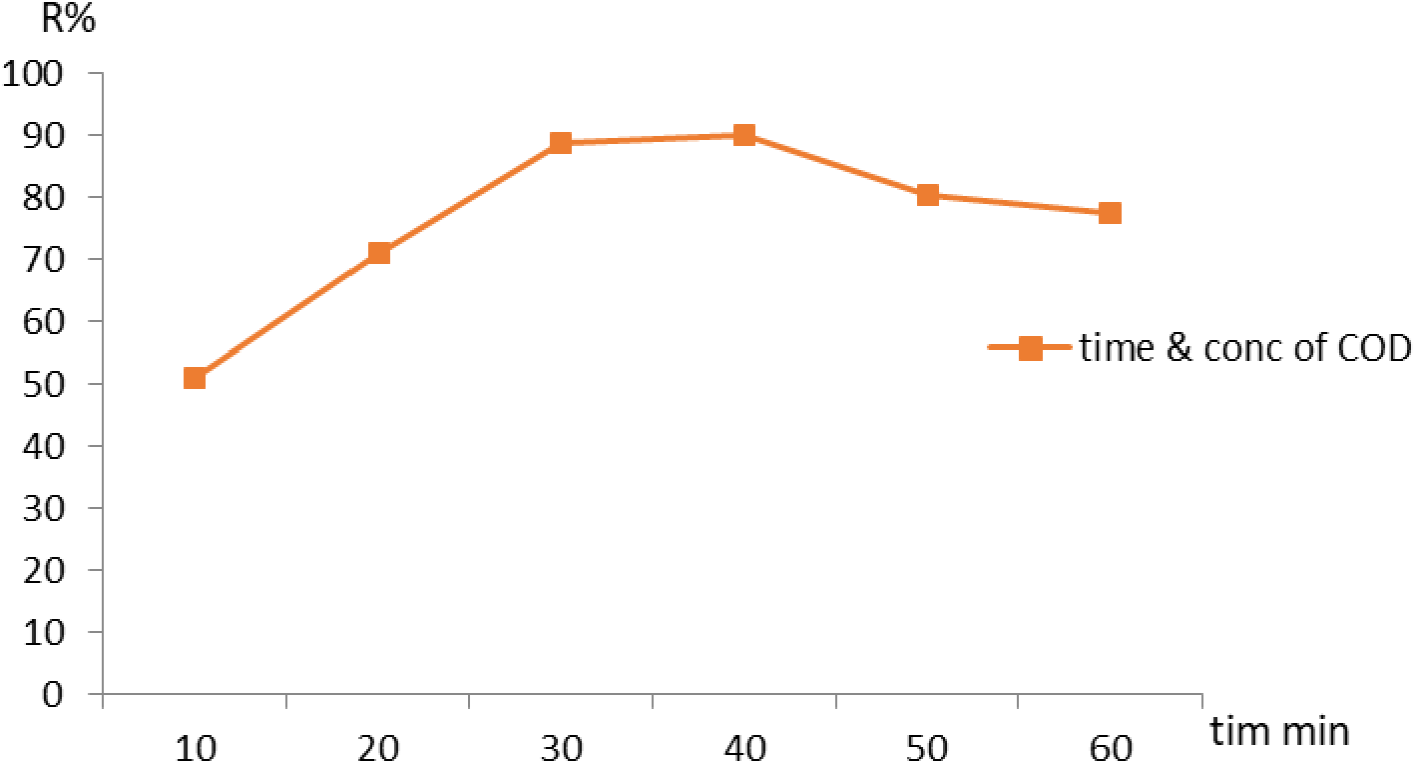
Adsorption of COD onto NaAZ as affected by the contact time at the optimum operating conditions.

During varied contact times the removal of COD by NaAZ was investigated. The studies were conducted at 20 °C. The adsorbent dose was 0.1 g, and contact times were 10, 20, 30, 40, 50, and 60 min. Fig 7 shows how contact time affects the adsorption of COD under the given conditions. The adsorbent was combined with a synthetic aqueous solution at a concentration of 500 mg/L [12]. The retention time that water stays in the treatment system, plays a role in determining the exposure time of pollutants to NaAZ. Higher retention time increase the likelihood of pollutants being removed, but also increase the size of the required system. The efficiency of pollutant removal generally increases with longer contact time, as the zeolite has more opportunity to interact with and adsorb the contaminants. Over time, the adsorption sites on NaAZ can become saturated, and beyond a certain point, increasing the contact time will not significantly increase the removal efficiency. Longer contact times generally lead to higher pollutant removal efficiencies because it allows more time for adsorption and ion exchange. Contact time refers to the amount of time the wastewater is in contact with NaAZ, allowing pollutants to adsorb or exchange with the zeolite surface. However, in practical applications, this effect typically occurs after a sufficient period of treatment. Less efficient removal, as the pollutants do not have enough time to diffuse into the zeolite structure and interact with the exchange sites. Higher pollutant removal efficiency, especially for pollutants that require more time to exchange or adsorb onto the NaAZ. The results showed that 40 min was the best duration for employing NaAZ to remove COD at removal efficiency is 90%.

### Effect of dose on adsorption profile

**Fig 8:**
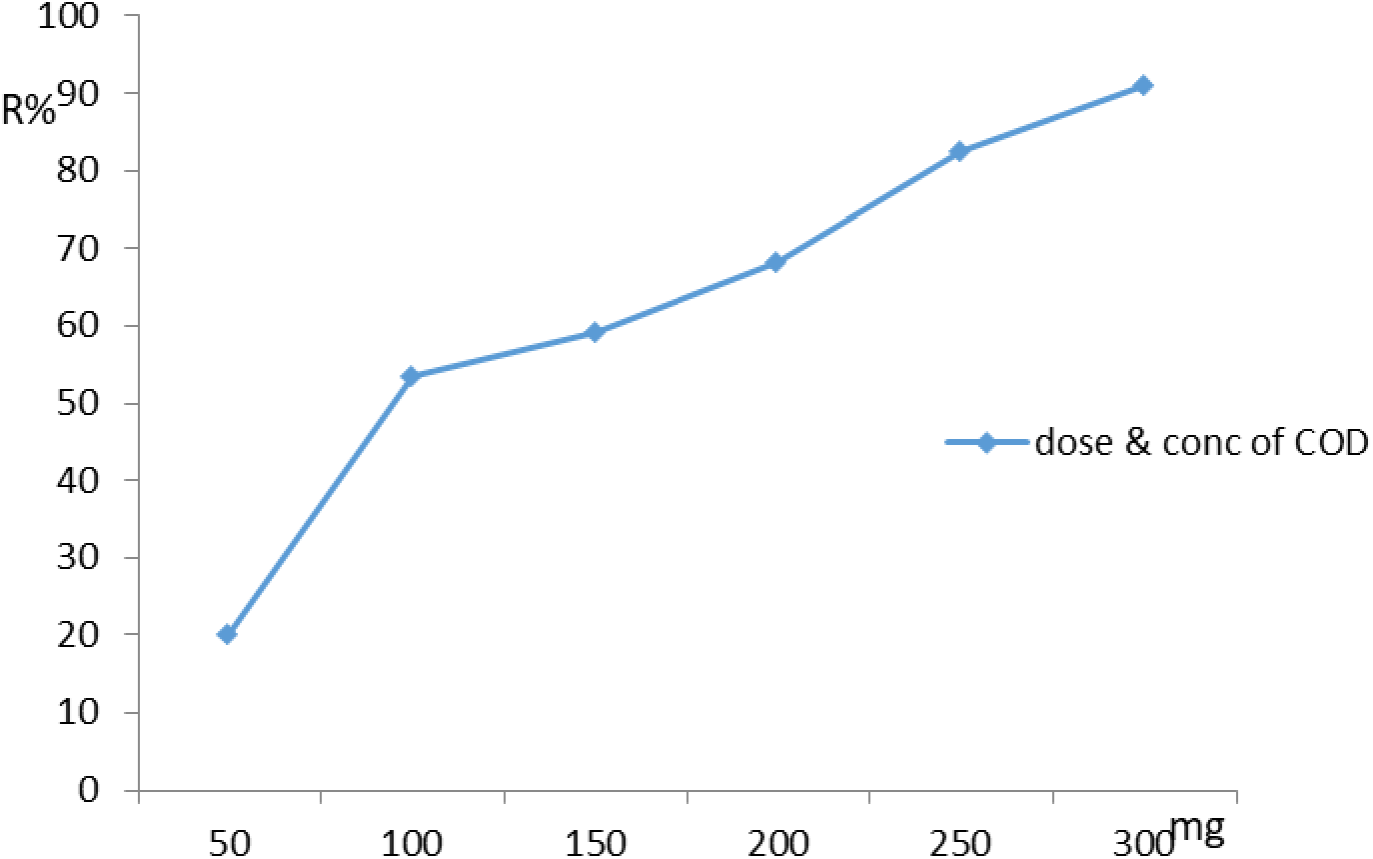
Influence of the adsorbent dose NaAZ on removal of COD at the optimum operating conditions.

The investigation of the effect of adsorbent dose on adsorption capacity is also part of batch studies. The aqueous solution volume utilized in the experiment was 1.0 L at 150 rpm, the contact period was 40 min, and the initial concentration of COD was 500 mg/L. The adsorbent was mixed with an aqueous solution at various dosages (0.05, 0.1, 0.15, 0.2, 0.25 and 0.3 g) in the combined system [12]. Insufficient adsorption sites leading to lower removal efficiency, especially for highly concentrated contaminants. Increasing the dose of NaAZ increases the number of available adsorption sites, which enhances the overall pollutant removal capacity, sites are available to adsorb salts, organic pollutants, ammonium ions, and other contaminants. After a certain point, increasing the dose further results in diminishing returns, where additional NaAZ provides little or no improvement in removal efficiency. The optimal Na A, zeolite dose balances pollutant removal efficiency and the cost of the material. Beyond the optimal dose, the extra NaAZ may not significantly enhance treatment, and it increases the cost of treatment unnecessarily. The removal effectiveness the dosage of NaAZ shows, the removal efficiency increased from 20% to 91% at dose 0.05 g to 0.3 g respectively, as shown in Fig 8.

### Effect of initial concentration

**Fig 9:**
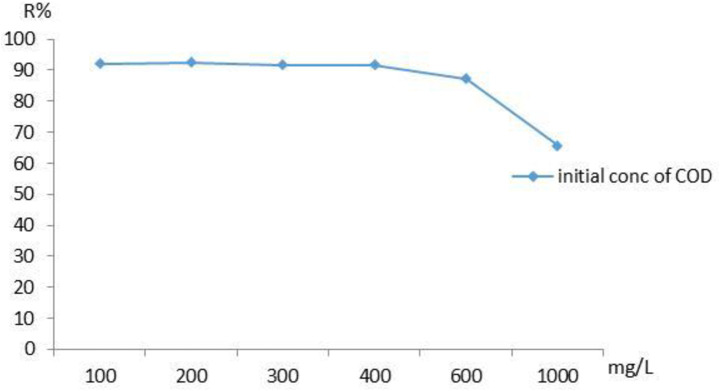
Influence of starting COD level on removal efficiency using NaAZ at optimum operating conditions.

The experiment was conducted by creating different COD concentrations and holding the temperature constant at 20 °C. It was investigated how the initial concentration of COD affected the effectiveness of adsorption [13]. Fig 9 shows the influence of starting levels (100, 200, 300, 400, 600 and 1000) mg/L with the same 40 min contact time and dose of 0.3 g. Fig 9 shows the influence of starting levels COD on the efficiency of the adsorption process. When increasing the starting levels of COD the percentage of adsorption capacity of NaAZ decreased from 92% to 65%. The aforementioned results seem to be mostly caused by the adsorbent’s limited number of active sites on its surface [13].

### Effect of pH

**Fig 10:**
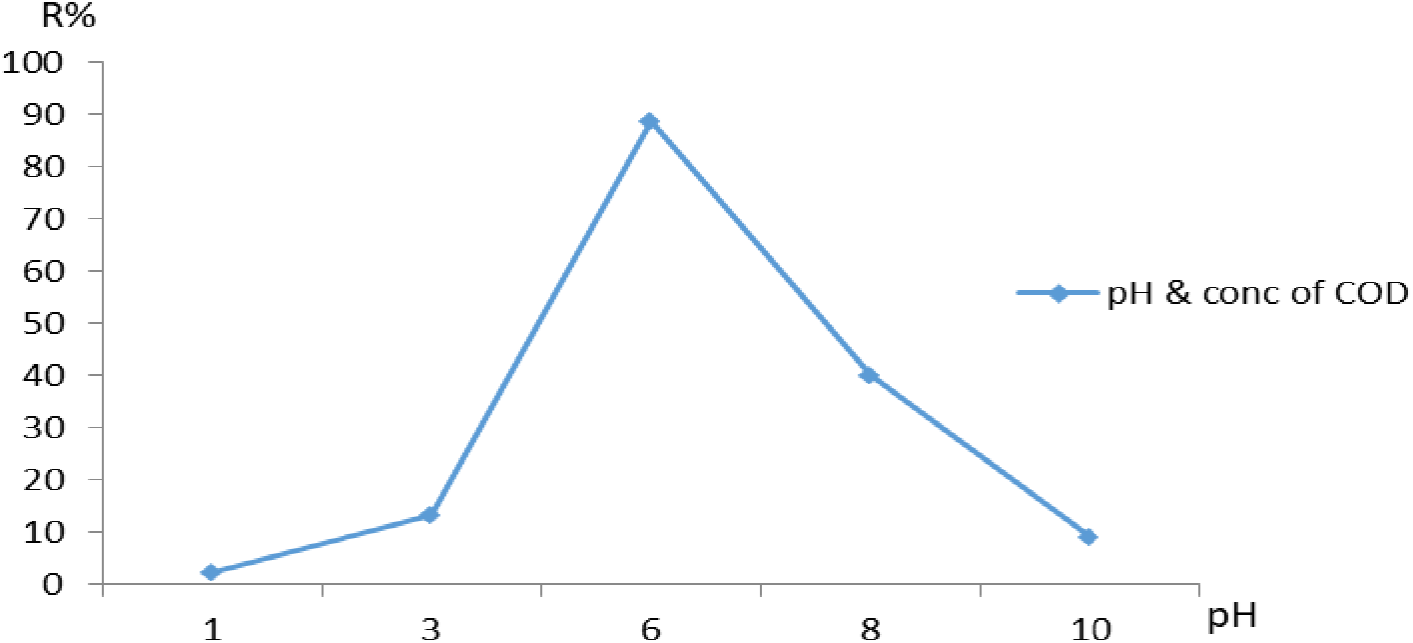
Adsorption of COD onto NaAZ as affected by the pH value at the optimum operating conditions.

The pH is the most critical parameter affecting any adsorption studies due to their interference in the solid–solution interface, affecting the charges of the active sites of the adsorbents and the COD behavior in the solution [13]. The effect of pH on the removal of COD in the solution has been established and considered an important parameter affecting the performance of the adsorption process. At varied pH levels (3, 6, and 9), removing of COD from the aqueous solution was conducted with a constant dosage of 0.1 g at 150 rpm/40 min, and 500 mg/L(12). The **pH** of the COD significantly influences the adsorption of ions onto Na A. zeolite. At very high pH levels, the presence of hydroxide ions (OH⁻) can hinder pollutants adsorption by precipitating out as hydroxides, reducing the efficiency of NaAZ in removing pollutants. The optimal pH for NaAZ generally falls in the slightly acidic to neutral range pH (6,7). At this pH range, NaAZ can efficiently adsorb both cations. At acidic pH pH (3): Better adsorption of COD but potentially less efficient ammonium removal. At neutral pH (pH 6–7): Optimal for pollutants. Fig 10 demonstrates that the highest COD removal occurred at an acidic pH of 6–7.

### Breakthrough curve in adsorption studies

**Fig 11:**
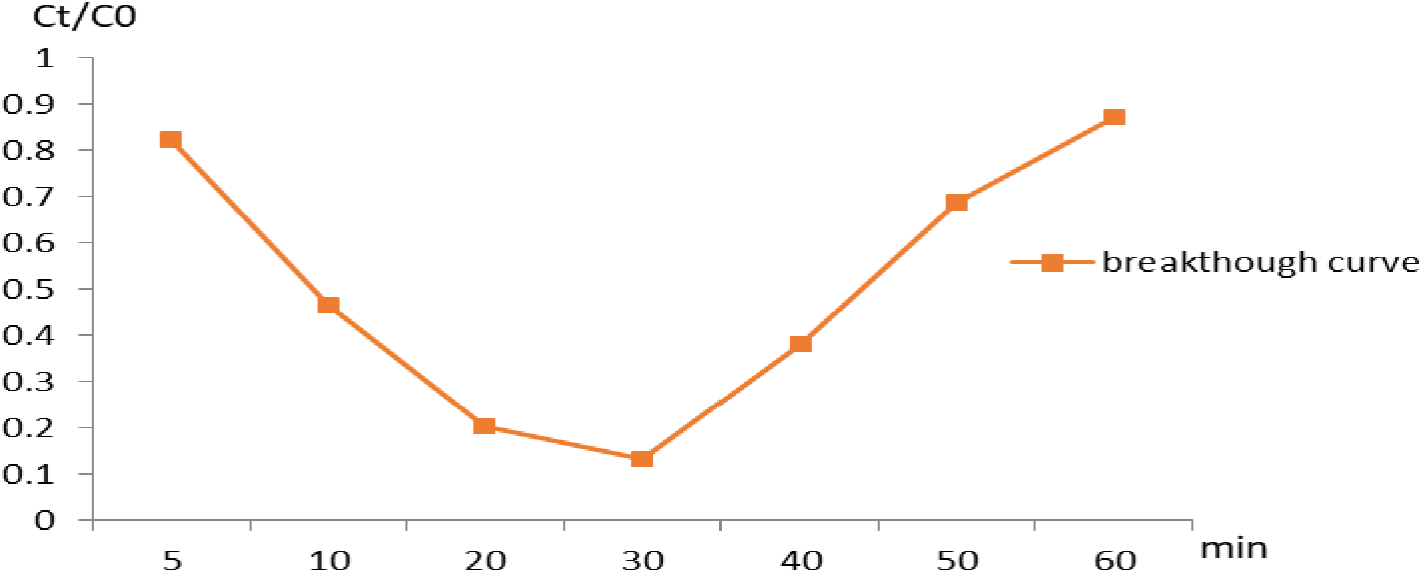
Breakthrough curve of study for NaAZ for removal COD.

Column studies simulate COD treatment systems and provide insights into the long-term performance and reusability of Na A. zeolite. Pack a column with Na A. zeolite. Pass contaminated water through the column at a controlled flow rate. Monitor the effluent for concentrations of pollutants. Measure the removal efficiency at different time (5, 10, 20, 30. 40, 50, and 60) min [14], factors affecting breakthrough curve on removal of NaAZ such as surface area, bed height, and flow rate of Cod solution. Flow rate of sample in column system is 2.5 ml/min, bed height (50 cm). The effectively adsorption of NaAZ for COD was plotted as C_t_/C_₀_ vs. time (t), where **C**_t_ = Effluent COD concentration (mg/L), **C**₀ = Initial COD concentration (mg/L). Fig 11 demonstrates that the highest COD removal occurred at 30 min.

### Regeneration and reusability studies of NaAZ

Regenerating zeolite allows its reuse for multiple cycles in wastewater treatment while maintaining high adsorption efficiency. Various studies and practical applications have demonstrated the effectiveness of regenerated zeolite in removing contaminants such as COD. Over time, zeolite becomes saturated reducing its efficiency; **r**egeneration is the process of restoring its adsorption capacity, allowing repeated use and improving cost-effectiveness. To assess the economic feasibility of NaAZ for wastewater treatment, studies on its regeneration and reusability are essential. This provides an understanding of the cost-effectiveness and sustainability of NaAZ in wastewater treatment applications. Regenerate the zeolite after a five cycles by flushing the column with a regeneration solution HCl (1.0 N). A five-cycle regeneration study evaluates the adsorption capacity as shows in Table 3, efficiency loss, and structural stability of zeolite when treating COD pollutants. The Non-significant degradation due to desorption with NaOH at ambient temperature effectively removes adsorbed species without attacking the zeolite framework, A high crystallinity index, and Sodium cations in the pores exchange reversibly with contaminants during adsorption and are readily restored upon washing.

**Table 3:** Performance evaluation over five cycles.

Cycle Number	Regeneration Efficiency (%)	Observation
1	76	Non-significant degradation
2	55.8	Slight efficiency drop, some pore clogging
3	27.6	Minor ion-exchange site reduction
4	11.1	Adsorption rate slows, partial pore collapse
5	2	Non- significant degradation

### Adsorption isotherms

**Fig 12:**
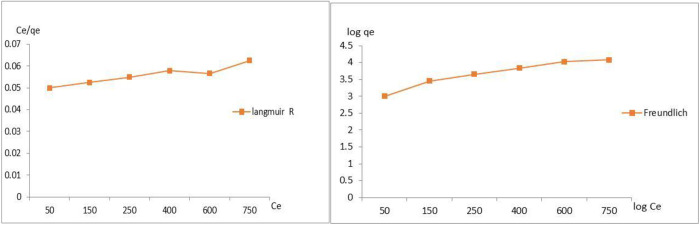
Langmuir and Freundlich isotherm models for COD on NaAZ.

The analysis of adsorption isotherms reveals important information, such as maximum adsorption capacity (Q, mg/g) and the adsorption mechanism, which can be used in the development of adsorption systems for wastewater treatment. The isotherm models are represented by curves that relate the amount of adsorbate at the adsorbent surface and the concentration of the adsorbate in the liquid phase. Table 4 displays the results for the Langmuir and Freundlich isotherms for the adsorption of COD on Na A. zeolite. The Freundlich isotherm model clearly outperforms the Langmuir isotherm model in terms of correlation coefficient (R^2^ > 0.90). This outcome shows that the Freundlich model and the experimental results have a good degree of agreement. Additionally, it appears from Fig 12 that the obtained data may be aligned with the Freundlich model. This model contends that the active sites are uniformly distributed across the adsorbent surface and that COD are adsorbed on NaAZ in a multilayer fashion. Table 4 shows the existing adsorption isotherm models used to characterize the adsorption on microporous materials. The models most commonly adopted to represent the adsorption isotherms are the Langmuir and Freundlich models, which assume a uniform distribution in monolayers at the adsorbent surface and the multilayer adsorption on a heterogeneous surface of the adsorbent, respectively [1].

**Table 4:** The isotherm adsorption parameters for the studied COD adsorbed on NaAZ.

Parameter	Langmuir isotherm model	Freundlich isotherm model
R^2^	0.9106	0.9277
K constant	0.045	644
N	—	4.8

### Kinetic model

**Fig 13:**
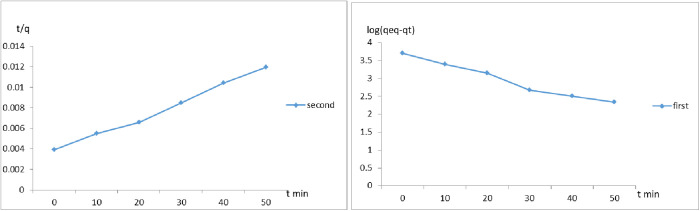
Kinetic models for COD adsorbed on NaAZ.

The rate of adsorption generally follows **a** pseudo-first-order kinetic model, meaning that the rate of adsorption decreases as the contaminant concentration decreases, reaching a point of equilibrium. Table 5 lists the constant values for the kinetic models for the adsorption of COD NaAZ as well as the related regression coefficients (R^2^). The adsorption kinetics of COD on NaAZ was 0.9944, and 0.9767 for second order model and first order model respectively, the curves shown in Fig 13, the study of data from the pseudo-second-order pattern leads to a conclusion that chemisorption regulates the adsorption of NaAZ. Hence, it is clear that the pseudo-second-order model best describes the kinetics of NaAZ. Based on the error analysis using the SSE, the pseudo-second-order kinetic model exhibited the lowest SSE value among the evaluated models. This indicates a superior fit between the model-predicted and experimental data, confirming that the adsorption process follows pseudo-second-order kinetics, which suggests that chemisorption may be the rate-limiting step involving valence forces through sharing or exchange of electrons between adsorbent and adsorbate.

**Table 5:** The adsorption kinetic models’ parameters regarding the of COD adsorbed on NaAZ.

Parameter	Second order model	First order model
R^2^	0.9944	0.9767
K constant	0.001219	0.123391
q_cal_	625	7988
q_exp_	454	

### Case study of using NaAZ in wastewater treatment

#### Characteristics of real raw wastewater.

By measurements of raw wastewater characteristics, it was found that the COD ranged from 470 to 473 mg/L. The TSS ranged from 244 to 275 mg/l, respectively. The turbidity ranged from 87 to 128 NTU. PO_4_^3-^ in wastewater varied from 6.1 to 7.5 mg/L. The samples measured in this study were ten samples and the test was repeated three times. The results of raw wastewater compared with law:48/1982 for discharge of the wastewater. These results are shown in Table 6. It was found that the wastewater is residential strength where the TSS lowers than 350, and pH from 6 to 9. Optimal conditions were applied to wastewater samples: a contact time of 40 minutes, a dosage rate of 250 mg, and a pH of 6–7 at room temperature of 20–25°C. The results obtained were used to determine the efficiency of the adsorbent and calculate the treatment ratio according to the equation. By applying the optimum factors to the wastewater samples, the treatment ratio was as follow COD 90.69%, TSS 90.41%, TKN 73.75%, Turbidity 85.05%, PO_4_^3–^68.85%^-^, and the treated samples fully conformed to the wastewater limits, as shown in Table 6.

**Table 6:** Characterization of raw wastewater.

Parameter	Unit	Average of pollutants in raw wastewater	Average of pollutants in treated wastewater	standard deviations (SD)	Removal percent %	law:48/1982 Secondary treated water
pH	—	6.75	7.33	0.7918485	—	6.00 - 9.00
EC	µs/cm	1286	1333	31.031597	—	2500-7500
TDS	mg/L	778	773	9.4318609	—	2000
Turbidity	NTU	87	13	1.4696938	85.05	—
COD	mg/L	473	44	12.302845	90.69	80
TSS	mg/L	240	23	4.4678406	90.41	40
PO_4_^3-^	mg/L	6.1	1.9	0.9334152	68.85	2.0
TKN	mg/L	14.1	3.7	0.1720465	73.75	5.0

ND is Not Detected; NTU is Nephelometric Turbidity Unit, average for ten samples.

#### Discussion on the removal of pollutants from wastewater Using NaAZ.

NaAZ has been found to remove up to 90.69% COD, NaAZ removes organic pollutants mainly through physical adsorption, where organic molecules from wastewater adhere to the surface of the zeolite due to van der Waals forces, hydrogen bonding, and dipole interactions. This leads to a reduction in the concentration of dissolved organic matter, which in turn lowers COD values, this comply with Freundlich isotherm model that a confirm for physical adsorption mechanism response for remove of COD from wastewater using NaAZ. The ion-exchange properties of NaAZ can also contribute to the removal of dissolved ions, such as ammonium (NH₄⁺) and TKN, which can indirectly influence the organic content by improving the overall water quality and promoting biological treatment processes. NaAZ has been found to support microbial growth. The zeolite’s porous surface can provide a habitat for microorganisms that degrade organic pollutants.

NaAZ has been found to remove up to 73.75% **of** TKN, ammonium adsorption due to the greater protonation of ammonia into ammonium ions. Ammonium ions (NH₄⁺) are common nitrogen-based pollutants in wastewater, excessive ammonia in wastewater can lead to eutrophication, algal blooms, and oxygen depletion in aquatic ecosystems. NH₄ ⁺ ion can be exchanged with Na⁺ in NaAZ. When NH₄ ⁺ -rich wastewater comes into contact with Na-zeolite, NH₄ ⁺ are exchanged with Na⁺ from the zeolite structure, this comply with EDX characterization for Na zeolite after treatment methods that confirm for removal of N pollutants from wastewater using NaAZ. The selectivity of NaAZ for NH₄ ⁺ ions over other cations (like sodium and potassium) makes it an efficient medium for nitrogen removal in wastewater treatment, and the NH₄ ⁺ which is positively charged, readily exchanges with Na⁺ present in the zeolite structure.

NaAZ has been found to remove up to 68.85**% of** phosphate ions (PO₄^3^⁻), Lower pH conditions (acidic) favor ammonium adsorption due to the greater protonation of ammonia into ammonium ions. Phosphate ions (PO₄^3^⁻) are negatively charged, which allows them to interact with the positively charged exchange sites within the zeolite structure. NaAZ primarily removes phosphate ions through cation-exchange mechanisms, though phosphate removal may not be as efficient as for ammonium or heavy metals.

#### Reduction of pathogenic bacterial count after subjected to NaAZ.

Zeolites are naturally occurring or synthetic alumino-silicate minerals with a porous structure that can adsorb various substances, including bacteria. Their application in reducing bacterial counts can be effective, but the effectiveness may vary between Gram-positive and Gram-negative bacteria due to differences in their cell wall structures. Comparative between Gram-positive bacteria and Gram-negative bacteria is shown in Table 7, there are different in the cell structure. Peptidoglycan and teichoic acid make up the thick cell wall of Gram-positive bacteria. Five glycines are used to cross-link the peptide chain between peptidoglycans. The cell wall of Gram negative bacteria, however, has a multilayer structure that is composed of a thin peptidoglycan, lipoprotein, cortical, phospholipid and lipopoly saccharide from the inside out. Gram-negative bacteria have a different peptidoglycan structure than Gram-positive bacteria because their peptidoglycans directly cross linked. Due to differences in their cell wall structures, Gram-positive and Gram-negative bacteria respond differently to certain antibiotics [32]. Due to electrostatic contact, negatively charged bacterial cells can interact with positively charged AgNPs (1) because bacteria to develop“pits”in their cell walls, (2) these pits allow bacteria to penetrate the periplasm. (3) Rip the cell membrane apart. (4) DNA condensing; (5) combining and coagulating with bacterial cytoplasm; (6) cytoplasmic component leaking. (7) Induce DNA condensing, resulting in DNA breakdown and lack of replication, which inhibits bacterial growth [33–37]. Gram Positive Bacteria, Have a thick peptidoglycan layer and no outer membrane, making them generally more susceptible to certain antibiotics and antimicrobial agents, [34,35], Gram Negative Bacteria, Have a thin peptidoglycan layer and an outer membrane that can provide a barrier to certain substances, making them more resistant to some antimicrobial treatments [37].

**Table 7:** Comparative summary of effects.

Bacterial Type	Effect of Na A. zeolite	References
Gram-Positive	Significant reduction noted	[38,39]
Gram-Negative	Moderate to significant reduction, depending on strain	[40,41]

**Fig 14:**
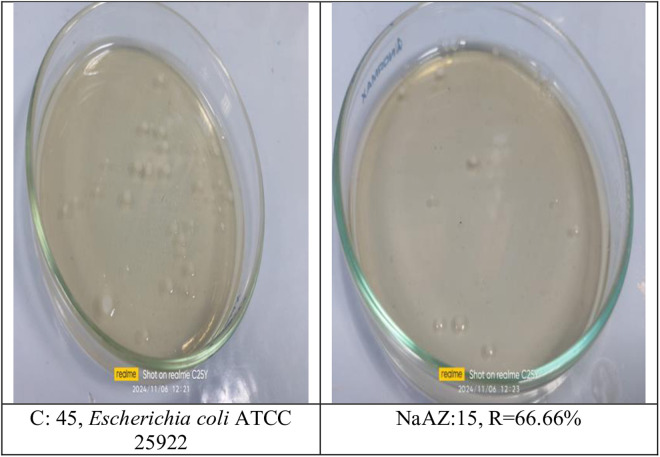
Escherichia coli reduction %.

**Fig 15:**
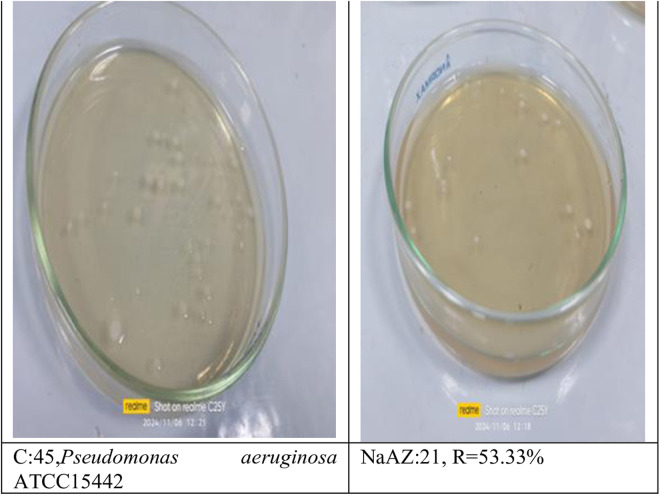
Pseudomonas aeruginosa reduction %.

**Fig 16:**
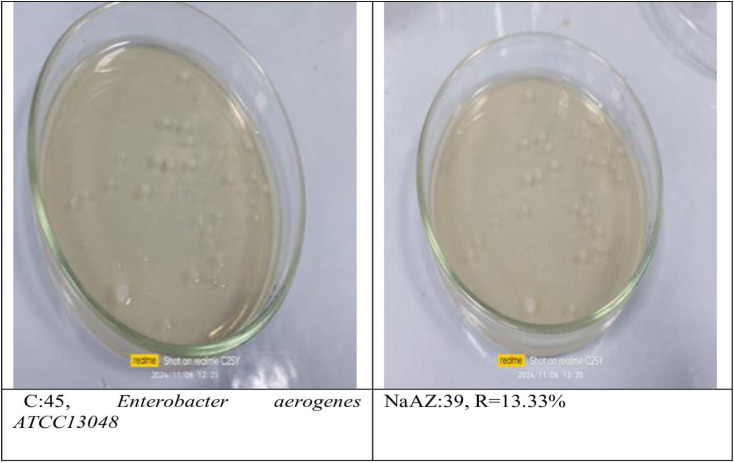
Enterobacter aerogenes reduction %.

**Fig 17:**
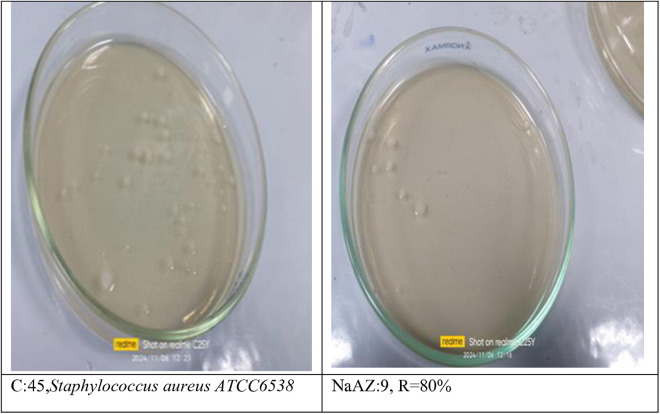
Staphylococcus aureus reduction %.

**Fig 18:**
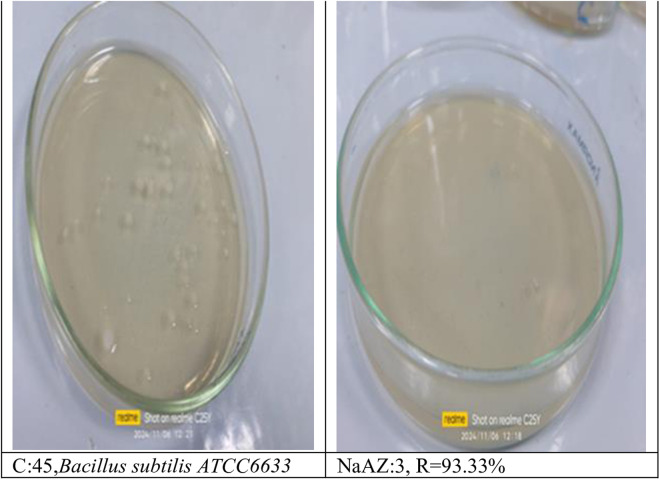
Bacillus subtilis reduction %.

**Fig 19:**
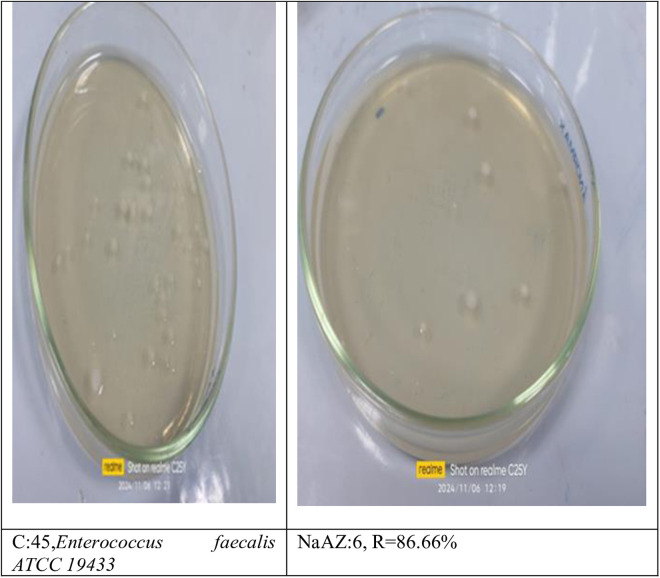
Enterococcus faecalis reduction %.

From our results it is clear that our tested samples NaAZ have highest antibacterial activites against gram positive bacteria than gram negative bacteria where highest count reduction occurred at *Bacillus subtilis* 93.33*10^4^ *Staphylococcus aureus* 75*10^4^ *and Enterococcus faecalis* 86.66*10^4^ as follow in Figs 14–19, and Table 8. It was clear from the results that the lowest value for count reduction obtained at *Pseudomonas aeruginosa gram negative with* 53.33%*10^4^.this result agrees with [26,42]. Generally more susceptible to zeolite treatment due to their exposed peptidoglycan layer, they may adhere more readily to zeolite surfaces, leading to higher reductions in bacterial. Gram-positive bacteria often show a greater sensitivity to natural zeolites because their thicker peptidoglycan layer allows better penetration of zeolite particles.

**Table 8:** Reduction of pathogenic bacterial count after subjected to NaAZ.

Control Bacterial inoculum 10^5^	Na A. zeolite	Reduction %
*Bacillus subtilis ATCC6633*	3*10^4^	93.33*10^4^
*Staphylococcus aureus ATCC6538*	11*10^4^	75*10^4^
*Enterococcus faecalis ATCC 19433*	6*10^4^	86.66*10^4^
*Escherichia coli ATCC 25922*	15*10^4^	60%*10^4^
*Enterobacter aerogenes ATCC13048*	39*10^4^	13.33%*10^4^
*Pseudomonas aeruginosa ATCC15442*	21*10^4^	53.33%*10^4^

Studies indicate that zeolites can reduce counts of various Gram-positive bacteria such as *Staphylococcus aureus* and *Bacillus cereus [*36*]*, May show some resistance due to their outer membrane, which can impede the interaction between the zeolite and the bacteria. However, zeolites can still have a significant effect through mechanisms like ion exchange and adsorption.

Gram-negative bacteria are often more resilient due to their outer membrane, which provides a barrier to many substances. However, certain zeolites have shown efficacy in reducing the populations of Gram-negative organisms such as *Escherichia coli* and *Salmonella spp [*36*]*. A study has reported that the application of zeolite in animal feed demonstrated a significant reduction of *Staphylococcus aureus* in the gut microbiome, enhancing overall health [38]. Studies Another recent investigation showed that zeolite can effectively reduce *Listeria monocytogenes counts* in food products [39]. A recent study found that natural zeolites significantly decreased the counts of *Escherichia coli* in wastewater treatments [41]. Research has indicated that zeolite treatment can mitigate *Salmonella* contamination in poultry, demonstrating a reduction in viable counts post-treatment [40]. Zeolite can effectively reduce bacterial counts for both Gram-positive and Gram-negative bacteria, but the degree of effectiveness may be higher for Gram-positive bacteria due to their cell wall structure [35–41].

### Toxicity test for NaAZ

NaAZ and silver nitrates (AgNO3) as the control were each added to 1.0 L of autoclaved distilled water (Devoid of ions bacteria, and other contaminants and prevents mineral deposits from accumulating within the autoclave chamber and on sterilized devices). The test was then conducted, and the results were reported in accordance with (ISO 11348–3:2018) employing the tested microbes *Vibrio fischeri* and microtox analyzer 500 is shown in Table 9. During testing, the bacteria were exposed to different Solutions, Along with standard solutions and control samples. The bacteria’s reduction in light emission was assessed using the microtox Omni Azur software, the data were recorded and the EC_50_ (concentrations generating a 50% reduction in light) is computed [20–22].

**Table 9:** Toxicity degrees and EC_50_ according to ISO 11348-3:2018 for (a) Silver nitrates (AgNO_3_) as control; (c) NaAZ.

Degree of toxicity	Extremely toxice	Very toxic	Toxic	Moderate toxic	Non toxic
EC_50_: value	0–19	20–39	40–59	60–79	80–100

EC_50_: The effective concentration causing 50% luminescence inhibition Bacteria.. Storage temperature: −25°C Reference substances used: 3,5- Di-chloro-phenol, Zinc sulfate hydrate, Potassium dichromate.

Toxicity Table 10: Effective concentrations and toxicity degrees for the three tested solutions, the toxicity effective concentration (EC) of the solutions that result in 50% inhibition in Bioluminescence of *Vibro Fischeri* (EC_50_) values were calculated after 30 min exposure time and at 15°C. The Microtox equipment includes a self-calibrating analyzer which incorporates a photomultiplier tube, a data collection and reduction system, and software. The temperature-controlled analyzer maintains the test organisms and samples at a standard temperature of 15°C. It also detects the light intensity at 490 nm, the wavelength emitted by the bacteria [43]. Vibrio fischeri is a bioluminescent, Gram-negative marine bacterium that can be found free living and in a mutualistic association with certain squids and fishes. This is a luminescent bacteria Vibrio fischeri used for in vitro test system Microtox®, which is applied for toxicity identification of pure or mixed chemicals and environmental samples [44].

**Table 10:** Effective concentrations and toxicity degrees for the three tested solutions.

Sample	EC_50_ (%)	Toxicity degree	PH before	PH after
Na A. zeolite	96	Non toxic	6.5	7.00
ZnSO_4_(control)	9	Extremely toxic	7.00	7.00

*Vibrio fischeri* are nonpathogenic, marine, luminescent bacteria which are sensitive to a wide range of toxicants. The organisms are supplied for use in a standard freeze-dried (lyophilized) state, which serves to maintain the sensitivity and stability of the test organisms. Disruption of the respiratory process, by exposure to a toxicant affects the metabolic pathway that converts chemical energy via the electron transfer system of the bacteria to visible light [45]. According to *Vibro fischeri* bacteria that were more sensitive to the toxic materials, zinc sulphates ions was harmful. This resulted from microbial cells resistance to the solubility of effective concentrations and toxicity degrees for the three tested solutions Na A. zeolite. The EC_50_ indicated that the toxicity declined as activation reduction mechanism. This can be explained as that the bacteria *V fischeri* can be employed as a biosensor for quick and inexpensive detection of acute nonmaterial toxicity due to its sensitivity to chemical toxicants. Our findings were in agreement with those founding where the EC_50_ for zinc sulphate was 9 with an extremely poisonous degree compared with EC_50_ for NaAZ_100_ and toxicity unit (nontoxic) [45–47].

### Comparative analysis of pollutant removal efficiencies

A comparison of the present study with previously published research on pollutant removal from wastewater demonstrates the competitive performance of the synthesized NaAZ. The removal efficiencies and adsorption capacities observed align well with, and in some cases surpass. This suggests that the synthesized NaAZ exhibits high potential as an effective and sustainable adsorbent. Table 11 is shown a comparative analysis including COD, TKN, TSS, TP, and Turbidity removal efficiencies using NaAZ in this study versus those from existing literature. The results from this study show that NaAZ exhibits comparable removal efficiencies for a wide range of wastewater pollutants. In particular, the high turbidity and TSS removal values demonstrate its strong particulate retention capability, while effective COD and TKN removal indicate strong adsorption of organic and nitrogenous compounds. The moderate yet promising TP removal efficiency suggests potential for further optimization or surface modification. The antimicrobial activity of NaAZ was assessed against a spectrum of pathogenic bacteria, including Gram-positive strains (*Bacillus subtilis*, *Staphylococcus aureus*, *Enterococcus faecalis*) and Gram-negative strains (*Escherichia coli*, *Enterobacter aerogenes*, *Pseudomonas aeruginosa*). Notably, a significant reduction in pathogenic bacterial counts was observed, confirming the dual functional performance of NaAZ as both an antimicrobial and an adsorbent material. Compared to other studies, such as *Mitra et al. (2018)* who reported inhibition zones of 12–15 mm for *S. aureus* and 10–13 mm for *E. coli* using Ag-loaded natural zeolite, who achieved 75–80% bacterial reduction using modified clinoptilolite, the NaAZ synthesized in this work demonstrated comparable or enhanced antibacterial efficiency, even without additional metal functionalization. This improved performance may be attributed to the highly crystalline nature, optimized pore structure, and effective ion-exchange capacity of the NaAZ, which collectively contribute to its ability to disrupt bacterial cell membranes and adsorb microbial contaminants from aqueous environments.

**Table 11:** A comparison of the present study with other studies on pollutant removal from wastewater.

Parameter	Removal efficiency % This Study (Na A Zeolite)	Removal efficiency % Literature Results
Turbidity	85.05	80%, Conventional filters, natural media [48*]*
COD	90.69	65 5, Natural zeolite, modified clays [37*]*
TSS	90.41	85%, Sand/gravel filtration, activated carbon [49]
PO_4_	68.85	60%, Modified zeolite, alum sludge [50]
TKN	73.75	63%, Clinoptilolite, natural zeolite [51]

## Conclusion

NaAZ was successfully synthesized through a controlled hydrothermal process involving sodium hydroxide, sodium meta-silicate, and aluminum sulfate as precursor materials. The formation of a gel followed by crystallization at 80°C for 12 hours resulted in a well-defined, granular zeolite structure after subsequent washing and drying steps. Overall, the NaAZ synthesized under these conditions not only showed promising structural and compositional properties but also exhibited effective antibacterial activity, making it a viable candidate for applications in water purification and microbial control. This study demonstrates the efficacy of NaAZ as an efficient adsorbent for wastewater treatment under optimized conditions—contact time of 40 minutes, pH 6–7, and a dosage of 30 mg. Adsorption studies indicated that the breakthrough curve was well-defined, and equilibrium data conformed best to the Freundlich isotherm model supported multilayer adsorption on heterogeneous surfaces, providing a broader understanding of the mechanism. Kinetic modeling showed that the adsorption followed the pseudo-second-order model, indicating chemisorption as the dominant mechanism. Comprehensive characterization using EDX, FT-IR, XRD, SEM, and BET confirmed the structural integrity, surface morphology, and porosity of the NaAZ, further validating its suitability for adsorption applications. Application of the optimized conditions to real wastewater samples resulted in significant removal efficiencies for key pollutants: COD (90.69%), TSS (90.41%), TKN (73.75%), and PO₄^3^⁻ (68.85%). Toxicity assessments confirmed the non-toxic nature of Na A. zeolite, while its in vitro antibacterial activity revealed strong efficacy against both Gram-positive (Bacillus subtilis, Staphylococcus aureus, Enterococcus faecalis) and Gram-negative (Escherichia coli, Enterobacter aerogenes, Pseudomonas aeruginosa) bacterial strains. Notably, a significant reduction in pathogenic bacterial count was observed, highlighting the dual functional role of NaAZ as both an adsorbent and antimicrobial agent. Compared to similar studies, the NaAZ synthesized in this work achieved high removal efficiencies across wastewater parameters including COD, TKN, TSS, TP, and turbidity highlighting its effectiveness as a versatile and sustainable material for integrated wastewater treatment applications. Overall, NaAZ presents a promising, multifunctional material for sustainable and effective wastewater treatment, combining pollutant removal with antibacterial properties under environmentally friendly conditions.

## Supporting information

S1 File Data.
(RAR)
